# Using core competencies to build an evaluative framework: outcome assessment of the University of Guelph Master of Public Health program

**DOI:** 10.1186/1472-6920-14-158

**Published:** 2014-07-31

**Authors:** Nicole Britten, Lauren E Wallar, Scott A McEwen, Andrew Papadopoulos

**Affiliations:** 1 Department of Population Medicine, Ontario Veterinary College, University of Guelph, Guelph, Canada

**Keywords:** Master of public health, Public health education, Graduate students, Program evaluation, Core competencies, Practicum, Curriculum map, Survey

## Abstract

**Background:**

Master of Public Health programs have been developed across Canada in response to the need for graduate-level trained professionals to work in the public health sector. The University of Guelph recently conducted a five-year outcome assessment using the *Core Competencies for Public Health in Canada* as an evaluative framework to determine whether graduates are receiving adequate training, and identify areas for improvement.

**Methods:**

A curriculum map of core courses and an online survey of University of Guelph Master of Public Health graduates comprised the outcome assessment. The curriculum map was constructed by evaluating course outlines, assignments, and content to determine the extent to which the Core Competencies were covered in each course. Quantitative survey results were characterized using descriptive statistics. Qualitative survey results were analyzed to identify common themes and patterns in open-ended responses.

**Results:**

The University of Guelph Master of Public Health program provided a positive learning environment in which graduates gained proficiency across the Core Competencies through core and elective courses, meaningful practicums, and competent faculty. Practice-based learning environments, particularly in collaboration with public health organizations, were deemed to be beneficial to students’ learning experiences.

**Conclusions:**

The Core Competencies and graduate surveys can be used to conduct a meaningful and informative outcome assessment. We encourage other Master of Public Health programs to conduct their own outcome assessments using a similar framework, and disseminate these results in order to identify best practices and strengthen the Canadian graduate public health education system.

## Background

High profile public health issues, such as the *Escherichia coli* contamination of the Walkerton, Ontario water supply in 2000, and the Severe Acute Respiratory Syndrome outbreak in the Greater Toronto Area in 2003, demonstrated the need for individuals with graduate-level public health training to improve the public health system. To meet this need, Master of Public Health (MPH) programs were developed by academic institutions across Canada. These universities, in partnership with public health organizations, seek to produce graduates that have the necessary skills, knowledge and attitudes to increase public health workforce capacity. However, the impact of MPH graduates on the Canadian public health system has not been formally assessed.

The development and continuous improvement of Canadian MPH programs are guided by two Public Health Agency of Canada documents: The *Core Competencies for Public Health in Canada* (Core Competencies) and the *Guidelines for MPH Programs in Canada* (Guidelines) [1,2]. The Core Competencies describe a set of 36 general competencies in 7 categories: public health sciences; assessment and analysis; policy and program planning, implementation and evaluation; partnerships, collaboration and advocacy; diversity and inclusiveness; communication; and, leadership [1]. The Guidelines advise all MPH programs to create a competency-based learning environment that clearly and systematically increases student proficiency across the Core Competencies through well-planned assignments, courses, and practicum experiences [2]. The Guidelines define a practicum as a “planned, supervised and evaluated field placement that provides the opportunity to integrate classroom learning and practice in a public health work environment” [2]. Public health competencies have previously been used to map preparedness training initiatives [3,4] and preventive medicine residency programs in the United States [5,6], as well as an MPH capstone assignment at the University of Guelph [7]. However, a knowledge gap exists in how to use the Core Competencies as an evaluative framework for Canadian MPH programs. Here, we describe the development and use of such a framework to conduct a five-year outcome assessment of the University of Guelph generalist MPH program. The goals of this assessment were to: 1) Assess whether the program is being delivered as intended based on current educational practices; 2) Assess whether the program is producing individuals adequately trained to begin careers in the public health sector based on their employability; 3) Provide accountability to the University of Guelph and the Canadian public health system; 4) Identify program strengths and weaknesses, as well as gaps in the intended curriculum; and, 5) Make recommendations for continuous improvement based on measures of graduate satisfaction, quality of practicum placements, and breadth and quality of courses.

## Methods

The Core Competencies were used as the framework for this two-part outcome assessment [1]. In the first part of the assessment, a curriculum map was created by the first author (NB), a recent graduate of the MPH program, to understand the distribution of the Core Competencies across the nine core courses. Course outlines, class assignments, and lecture material were reviewed to determine the extent to which the competencies were covered in each course. Each competency was rated using a five-point scale: 1 – No coverage, 2 – Minimal coverage, 3 – Moderate coverage, 4 – Substantial coverage, and 5 – Core component of the course. A competency that is minimally covered was briefly taught in lectures but was not needed to complete assignments. Moderate coverage indicates that a competency was taught in two or more lectures and was needed to complete assignments. Substantial coverage indicates that a competency was highly relevant to, but not a primary focus of, a course where it was taught in numerous lectures and needed to complete assignments. A competency that is a core component of a course was incorporated into the course learning objectives, taught throughout the entire course, and was directly assessed in course assignments. Competency 1.5 “Demonstrate the ability to pursue lifelong learning opportunities in the field of public health” was not included in the curriculum map as it reflects an ability that is developed throughout one’s professional career. The curriculum map was reviewed and validated by a second graduate of the MPH program, and the program coordinator, AP.

In the second part of the assessment, a logic model was first developed that identified the program’s inputs, activities, outputs, outcomes, impact and external factors (see Additional file 1). The logic model and the program’s critical success factors were used to develop survey questions. An online survey was constructed using the software program, LimeSurvey (http://www.limesurvey.org/en/). Informed consent was obtained prior to entering into the survey. The survey asked graduates to retrospectively assess their overall level of proficiency at the beginning of their program, and their proficiency in each of the Core Competencies at the end of their program, using a five-point scale: 1 – Needs improvement, 2 – Satisfactory, 3 – Good; 4 – Very good, and 5 – Outstanding. Students were also asked questions relating to the practicum placement, program curriculum and experience, and employment after graduation. In February 2013, the survey URL link was electronically distributed to all graduates via email and the University of Guelph MPH Facebook group page. Graduates were given three weeks to complete the survey.

Analysis of the survey results was done using Excel. Any incomplete surveys were excluded from analysis. Descriptive statistics were determined for all ratings scale questions. Time of graduation was cross-referenced with employment status to provide a standard frame of reference. Open-ended comments were reviewed to identify key themes and patterns for program improvement. This study was approved by the University of Guelph Research Ethics Board (REB 13JA055).

## Results

### Curriculum map

The curriculum mapping exercise revealed that all of the Core Competencies were covered by the nine core courses, at least to a moderate level (see Additional file 2). Thirty-three of the 35 Core Competencies were substantially covered, or were a core component of course material in one or more of the core courses. Competency 3.6 “Evaluate an action, policy, or program and competency” and Competency 3.8 “Demonstrate an ability to fulfill functional roles in an emergency” were moderately covered, at best, in all courses.

### Graduate outcome assessment survey

Of the 60 eligible participants, 44 submitted their responses (35 complete, 9 incomplete). The incomplete responses were excluded from analysis yielding a response rate of 58%.

### Employment

At the time of the survey, 60% of respondents were employed in the public health sector with the majority being employed for 3 years or less (Table 1). Of those employed in the public health sector, all were employed in 6 months or less with 71.4% being employed within 3 months. Of those who were not currently employed in the public health sector (40%), several respondents (30.8%) indicated that they were pursuing further education. Of those who were unable to find work (38.5%), 4 out of 5 of these respondents had graduated in February 2013.

**Table 1:** Descriptive statistics of survey respondents’ employment status, duration of employment, time to find employment, and reasons for unemployment in the public health sector

**Respondents employed in the public health sector (60%; 21 respondents)**			**Respondents not currently employed in the public health sector (40%; 13 respondents)**		
Duration of employment	Recent hire	14.3% (3)	Reason for not being employed in public health sector	Chosen a different career path	7.7% (1)
	<1 year	28.6% (6)		Pursuing further education	30.8% (4)
	1- 3 years	42.9% (9)		Unable to find work	38.5% (5)
	>3 years	9.5% (2)		Other	23.1% (3)
	No answer	4.8% (1)			
Time to find employment	<3 months	71.4% (15)			
	3-6 months	19.1% (4)			
	6 months- 1 year	0.00% (0)			
	> 1 year	0.00% (0)			
	No answer	9.5% (2)			

### Proficiency in the core competencies

When asked to evaluate their overall proficiency across the Core Competencies at the beginning of their program, 5.7% of respondents scored their proficiency as very good, none rated their proficiency as outstanding, with the majority of respondents needing improvement (40.0%). At the end of their program, the proportion of respondents who scored their proficiency as very good or outstanding in each of the Core Competencies ranged from 35.3-97.1% (see Additional file 3). Respondents felt least proficient in Competency 3.4 “Implement a policy or program and/or take appropriate action to address a specific public health issue” with 35.3% scoring their proficiency as very good (26.5%) or outstanding (8.8%). Respondents felt most proficient in Competency 6.2 “Interpret information for professional, non-professional and community audiences” with 97.1% scoring their proficiency as very good (50.0%) or outstanding (47.1%). The majority of respondents (greater than 50%) scored their proficiency as very good or outstanding in 31 of the 35 competencies. The other four competencies where less than 50% of respondents scored their proficiency as very good or outstanding were in Policy and Program Planning, Implementation and Evaluation (3.4, 3.5, 3.6, 3.8).

### Practicum placement

Students complete a 12-16 week practicum in the Spring/Summer semester as part of the MPH degree requirements. The majority of respondents (93.9%) rated the value of their practicum to their overall program experience as very good (36.4%) or outstanding (57.6%). Respondents indicated that the practicum experience added to their training by providing contact with individuals in the public health sector (82.9%), meaningful public health exposure (80.0%), ability to apply knowledge gained through coursework (80.0%), additional knowledge (80.0%), experience with challenges in the public health sector (71.4%), enhanced understanding of the value of the Core Competencies (57.1%), and other benefits (11.4%). Respondents indicated additional benefits including exposure to government public health agencies, and a greater understanding of public health infrastructure and communication hierarchies.

### Electives

In addition to the nine core courses, students must complete three restricted elective courses. The majority of respondents (60.0%) indicated that three electives in their area of interest was sufficient, whereas 22.9% would have liked to have taken more electives. When respondents were asked how well elective courses enhanced their knowledge and skills beyond that gained in the core courses, 75.8% of respondents scored their electives as very good (48.5%) or outstanding (27.3%). The most influential factor affecting selection of elective courses was general interest (82.9%), followed by course content (68.6%) and lacking knowledge and training in the course subject (60.0%) (Table 2). The least influential factor was a distance education learning format (11.4%). Additional comments revealed electives that are interdisciplinary, relevant to public health, and/or of personal interest were desirable. Course scheduling was cited as a challenge, at times, for choosing electives.

**Table 2:** Descriptive statistics of factors influencing elective selection including interest, lack of knowledge, course content, learning format, timing, and choice restriction

**Factors influencing elective choice**	**Percentage of respondents who selected the factor**
General interest	82.9% (29)
Knowledge and training in the course subject was lacking	60.0% (21)
Course content	68.6% (24)
Preference for distance education	11.4% (4)
Convenience/Time of delivery	31.4% (11)
Only option available	20.0% (7)
Other	11.4% (4)

### Faculty and program experience

Faculty are a central component of the MPH program. Faculty were rated on their level of student interaction, and teaching competence. The majority of respondents rated faculty as very good or outstanding in these areas (91.4% and 88.6%, respectively). Together, 85.7% of respondents rated their overall program experience as very good (25.7%) or outstanding (60.0%). Based on respondent feedback, key learning areas for future development include program evaluation, emergency preparedness, qualitative research skills, analytical software tools, and priority populations. More professional development opportunities such as networking and attending conferences would have further enhanced their development. Respondents identified practice-based learning environments, particularly in collaboration with public health organizations, as beneficial to their learning experience.

## Discussion

Most MPH programs in Canada were developed after a series of public health crises in the early 2000s exposed an overall lack of capacity in the Canadian public health system to meet population health needs, especially during times of crisis. Several government-commissioned reports that reviewed provincial and federal public health systems were unanimous in their recommendation for graduate-trained public health professionals [8-10]. In response, MPH programs were developed in order to imbue the necessary knowledge, skills and values in the future public health workforce. According to the Guidelines, MPH programs should provide students with a broad knowledge of concepts that can be applied to meet core public health needs [2]. This knowledge should be acquired in a competency-based learning environment that exists in both the classroom and real-world settings. In this study, we used the Core Competencies as the basis of our outcome assessment to gain a greater understanding of our competency-based learning environment. This allowed us to examine the progression of student learning across the Core Competencies, and to identify critical factors in student development that can be applied to similar MPH programs across Canada.

This study represents the first outcome assessment of a Canadian MPH program that uses the Core Competencies as an evaluative framework. The Core Competencies define the knowledge, skills and attitudes that are expected of today’s public health professionals [1]. Depending on training, experience and role in the public health system, public health professionals may develop greater proficiency in certain competencies. Ensuring that public health professionals acquire and maintain competence is a shared responsibility of the individual, educators, and the public health sector, with MPH programs providing the necessary skills and knowledge prior to entering the workforce.

The results of this assessment show that the University of Guelph MPH program graduates had a positive learning experience characterized by gains in proficiency in the Core Competencies, leading to rapid employment for those who entered the public health workforce. Key program factors included a comprehensive core curriculum, diverse electives, meaningful practicums, and interactive, knowledgeable faculty. Although our findings are specific to the University of Guelph MPH program, the evaluative framework used in this study can be similarly applied to other Canadian MPH programs in order to develop a systems-view of graduate public health education and training in Canada.

We found that the majority of respondents scored themselves as either needing improvement or having basic proficiency across the Core Competencies at the beginning of the MPH program. Given the diverse educational backgrounds and prior work experience of entering students, some variation in students’ level of proficiency can be expected. Over the duration of the program, students acquire the skills and knowledge necessary for public health professionals to become competent in their role, which are enhanced through practice-based learning experiences in order to make graduates proficient in the Core Competencies.

As students progressed through the program, we observed a shift where the majority of respondents scored themselves as having very good or outstanding proficiency in many of the Core Competencies at the end of the program. Given that the University of Guelph MPH program is a generalist program with no formal areas of specialization, students appeared to supplement their learning experience by choosing electives that enhanced their knowledge and training in areas of interest to them. Other critical factors in their development including meaningful practicum experiences, competency-based curriculum, and knowledgeable faculty were found to revolve around practice-based teaching experiences.

Practice-based teaching in public health unites the classroom and public health practice in order to enhance student learning [11]. The use of case studies and problem-based learning exercises in the classroom, and practicums outside of the classroom are common examples of practice-based teaching in public health [11,12]. Indeed, respondents identified the practicum experience as an extremely valuable component of their MPH program experience. Although there is little research on the impact of Canadian MPH practicums on student development, previous studies have shown that practicums can be successful in preparing students for entry into the workforce by developing additional skills, creating networking opportunities, and revealing what it is like to work in a public health setting that, together, help to bridge the gap between the school and work environment [13]. According to the Guidelines, MPH practicums allow students to apply their classroom knowledge to public health practice, enhance their proficiency in the Core Competencies, improve their interpersonal, communication, critical thinking and problem-solving skills, and identify potential career interests [2]. In order to provide valuable practicum experiences, successful links between academic institutions and public health practice must be established.

Linkages between academic institutions and public health practice allow the integration of traditional and applied learning in order to enhance student proficiency in the Core Competencies. A recent study of the partnership between New England boards of health and academic institutions found that boards of health were involved in hosting practicum students and in providing mentors, scholarly resources, guest lecturers, and financial support of student conference presentations [14]. In Florida, academic institutions support education and research within public health agencies, and in turn, public health agencies host MPH practicum students and serve as adjunct faculty [15]. Students enhanced epidemiological surge capacity in North Carolina through a partnership between the University of North Carolina Center for Public Health Preparedness and state and local health departments [12]. This kind of cooperative relationship was recommended by the Institute of Medicine’s “*Who will keep the public healthy? Educating public health professionals for the 21*^*st*^*century”* and is outlined in the Guidelines as a way to build both program and workforce capacity [2,16]. Although further research is needed to understand the synergistic effect of partnerships between Canadian MPH programs and public health agencies, these examples demonstrate that an effective workforce is a result of formal education and workplace learning. Incorporating practice-based learning in MPH pedagogical practices will accelerate this outcome.

Potential limitations of this study include recall bias, particularly for those respondents who were among the first cohort of graduates, and may have experienced some difficulty differentiating between knowledge gained while in the public health workforce, and their knowledge upon graduation. Response bias may have been introduced through self-selection or use of ratings scale questions. Of note, recent graduates (February 2013) were over-represented (28.6%) in the respondent population. Future work will evaluate employers’ perceptions of graduate proficiency in the Core Competencies to gain a more comprehensive understanding of the program’s impact and effectiveness. Although these results are limited to the University of Guelph MPH program, we encourage other MPH programs to use a similar evaluative framework, as presented in this study.

## Conclusions

Moving forward, a systems understanding of Canadian graduate public health education is needed and should include an evaluation of the impact of MPH graduates on the Canadian public health system. The public health workforce can benefit by identifying strengths and areas of improvement in MPH program curriculum, practice-based teaching, and linkages with public health agencies. We recommend that MPH programs conduct and publish regular evaluations and scholarly teaching and learning activities in order to identify and disseminate best practices for the strengthening of the entire public health education system. By using the Core Competencies as an evaluative framework, as presented here, results from different programs will be more easily translatable. As public health continues to face both new and recurring challenges, MPH programs will continue to be invaluable to the education and training of the public health workforce to meet these needs and enhance population health into the future.

## Abbreviations

MPH: Master of public health.

## Competing interests

The authors declare that they have no competing interests.

## Authors’ contributions

NB participated in the design of the study, analyzed the data, and drafted the manuscript. LEW analyzed the data, and drafted the manuscript. SAM participated in the design of the study, and helped to draft the manuscript. AP conceived of the study, participated in its design and coordination, and helped to draft the manuscript. All authors read and approved the final manuscript.

## Authors’ information

NB is a graduate of the University of Guelph MPH program. SAM is the admissions coordinator, and AP is the program coordinator of the University of Guelph MPH program.

## Pre-publication history

The pre-publication history for this paper can be accessed here:

http://www.biomedcentral.com/1472-6920/14/158/prepub

## Supplementary Material

Additional file 1 **Logic model of the University of Guelph MPH program outcome assessment.** The logic model describes the program inputs, activities, outputs, outcomes and impact, as well as influential external factors. Click here for file

Additional file 2 **Curriculum map of the nine core courses in the University of Guelph MPH program.** The Core Competencies were mapped onto the nine core courses using lecture material and course outlines and assignments. The extent to which they were covered in each course was scored using a 5-point scale: 1 – No coverage, 2 – Minimal coverage, 3 – Moderate coverage, 4 – Substantial coverage, 5 – Core component. Click here for file

Additional file 3 **Respondents’ Self-Assessment of their Proficiency in the Core Competencies upon Completion of the University of Guelph MPH Program.** Survey respondents rated their proficiency at the end of their program in 35 of the 36 Core Competencies using a 5-point scale: 1 – Needs improvement; 2 – Satisfactory; 3 – Good; 4 – Very good; 5 – Outstanding. Click here for file
