# Supplementary figures and images for: Using core competencies to build an evaluative framework: outcome assessment of the University of Guelph Master of Public Health program
Source: BMC Med Educ. 2014 Jul 31;14:158. doi: 10.1186/1472-6920-14-158 (PMC4131476; doi:10.1186/1472-6920-14-158)

## Additional file 1: Logic model of the University of Guelph MPH program outcome assessment

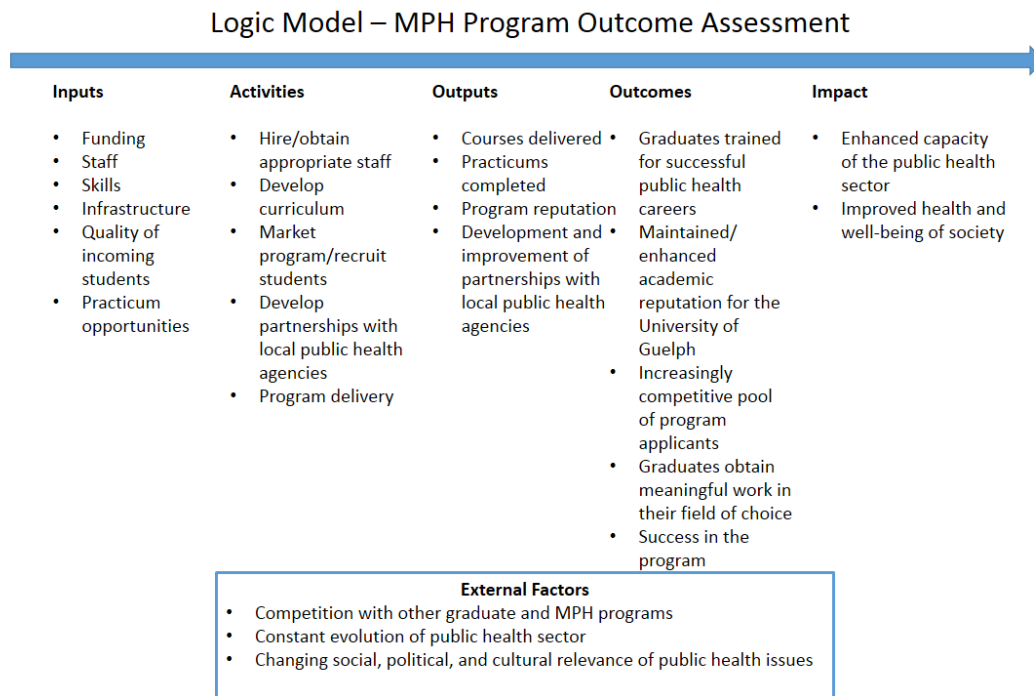

Supplement: Additional file 1 — Logic model of the University of Guelph MPH program outcome assessment. The logic model describes the program inputs, activities, outputs, outcomes and impact, as well as influential external factors. [file 1472-6920-14-158-S1.pdf]
