# Supplementary material for: Using core competencies to build an evaluative framework: outcome assessment of the University of Guelph Master of Public Health program
Source: BMC Med Educ. 2014 Jul 31;14:158. doi: 10.1186/1472-6920-14-158 (PMC4131476; doi:10.1186/1472-6920-14-158)
Supplement: Additional file 2 — Curriculum map of the nine core courses in the University of Guelph MPH program. The Core Competencies were mapped onto the nine core courses using lecture material and course outlines and assignments. The extent to which they were covered in each course was scored using a 5-point scale: 1 – No coverage, 2 – Minimal coverage, 3 – Moderate coverage, 4 – Substantial coverage, 5 – Core component. [file 1472-6920-14-158-S2.pdf]

Additional file 2: Curriculum map of the nine core courses in the University of Guelph MPH program

Scale: 1 – No coverage, 2 – Minimal coverage, 3 – Moderate coverage, 4 – Substantial coverage, 5 – Core component

	**POPM 6510 CHP**	**POPM 6200 Epi I**	**POPM 6520 EpiStats**	**POPM 6530 Comm I**	**POPM 6540 Enviro**	**POPM 6550 Policy**	**POPM 6570 Comm II**	**POPM 6580 Admin**	**PABI 6500 ID**
**1. Public Health Sciences**									
1.1 Knowledge of health status, inequities, determinants, health promotion strategies…	5	2	1	3	4	3	1	2	4
1.2 Knowledge about history & interaction of public health & health care	2	3	2	2	1	5	2	1	2
1.3 Apply public health sciences to practice	5	5	5	4	4	3	3	4	4
1.4 Use evidence & research to inform policy and programs	4	4	4	2	4	5	4	5	5
**2. Assessment and Analysis**									
2.1 Recognize a health concern or issue exists	4	4	4	2	4	3	2	4	4
2.2 Identify relevant and appropriate sources of information	4	2	4	1	4	4	1	2	4

2.3 Collect, store & use accurate & appropriate information	3	4	5	2	2	3	2	3	3
2.4 Analyze information to determine appropriate uses & gaps	3	5	4	2	3	3	1	3	4
2.5 Determine meaning of information considering contexts	3	2	2	3	3	4	4	4	5
2.6 Recommend specific action based on analysis of information	4	2	2	2	3	4	3	5	3
**3. Policy & Program Planning, Implementation & Evaluation**									
3.1 Describe selected policy & program options for specific issues	3	2	3	3	3	5	3	4	4
3.2 Describe implications of options & recommend course of action	4	2	2	3	3	5	2	3	5
3.3 Develop a plan taking relevant information into account	4	3	3	2	2	5	2	5	4
3.4 Implement a policy or program and/or take appropriate action	4	1	1	1	3	5	2	4	3
3.5 Demonstrate ability to implement effective practice guidelines	4	1	1	1	3	4	1	3	3

3.6 Evaluate an action, policy or program	2	1	2	1	2	3	1	3	2
3.7 Demonstrate ability to set & follow priorities, & max. outcomes	2	2	3	2	2	4	2	4	4
3.8 Demonstrate ability to fulfill functional roles in an emergency	1	3	2	3	3	2	1	3	3
**4. Partnerships, Collaboration and Advocacy**									
4.1 Identify & collaborate with partners	3	1	2	2	2	4	2	4	2
4.2 Use skills such as team building & conflict management to build partnerships	3	1	1	3	1	2	2	4	1
4.3 Mediate between differing interests & facilitate allocation of resources	2	1	1	3	2	4	2	4	3
4.4 Advocate for health public policies & services	4	1	1	3	3	4	1	3	3
**5. Diversity and Inclusiveness**									
5.1 Recognize how the determinants of health influence health of specific groups	5	2	2	3	3	3	2	4	5
5.2 Address population diversity when planning, implementing and evaluating programs & policies	5	2	2	4	3	4	2	3	4

5.3 Apply culturally-relevant & appropriate approaches with people of diverse backgrounds	5	2	2	4	3	4	3	4	4
**6. Communication**									
6.1 Communicate effectively with individuals, families, groups, communities & colleagues	4	3	3	5	3	4	5	4	4
6.2 Interpret information for professional, non-professional and community audiences	3	4	4	5	4	2	5	3	4
6.3 Mobilize individuals & communities by using appropriate media, resources & social marketing	4	1	1	5	3	2	5	3	5
6.4 Use current technology to communicate effectively	2	1	1	5	2	2	5	2	3
**7. Leadership**									
7.1 Describe the mission & priorities of the organization where one works, & apply them in practice	3	1	1	2	1	3	3	5	2

7.2 Contribute to developing key values & shared vision in implementing policy & programs	3	1	1	2	1	3	2	5	1
7.3 Utilize public health ethics to manage self, others, information & resources	3	1	3	3	2	4	4	4	4
7.4 Contribute to team and organizational learning to advance public health goals	2	3	3	3	3	4	4	5	3
7.5 Contribute to maintaining organizational performance standards	2	2	3	3	3	4	3	5	2
7.6 Demonstrate ability to build community capacity by sharing knowledge, tools, expertise & experience	4	4	3	4	3	3	5	4	4

*POPM 6510 – CHP = Community Health Promotion; POPM 6200 – Epi I = Epidemiology I; POPM 6520 – EpiStats = Introduction to Epidemiological and Statistical Methods; POPM 6530 – Comm I = Communications I; POPM 6540 – Enviro = Concepts in Environmental Public Health; POPM 6550 – Policy = Public Health Policy and Systems; POPM 6570 – Comm II = Communications II; POPM 6580 – Admin = Public Health Administration; PABI 6500 – ID = Infectious Diseases and Public Health
