# Supplementary material for: Using core competencies to build an evaluative framework: outcome assessment of the University of Guelph Master of Public Health program
Source: BMC Med Educ. 2014 Jul 31;14:158. doi: 10.1186/1472-6920-14-158 (PMC4131476; doi:10.1186/1472-6920-14-158)
Supplement: Additional file 3 — Respondents’ Self-Assessment of their Proficiency in the Core Competencies upon Completion of the University of Guelph MPH Program. Survey respondents rated their proficiency at the end of their program in 35 of the 36 Core Competencies using a 5-point scale: 1 – Needs improvement; 2 – Satisfactory; 3 – Good; 4 – Very good; 5 – Outstanding. [file 1472-6920-14-158-S3.pdf]

Additional file 3: Respondents' self-assessment of their proficiency in the Core Competencies upon completion of the University of Guelph MPH Program

Scale: 1 – Needs improvement; 2 – Satisfactory; 3 – Good; 4 – Very good; 5 – Outstanding.

**Competency**	**Score**					**n**
	**1**	**2**	**3**	**4**	**5**	
**1. Public Health Sciences**						
1.1a Demonstrate knowledge about the health status of populations, inequities in health, the determinants of health and illness	2.9%	2.9%	5.7%	57.1%	31.4%	35
1.1b Demonstrate knowledge about strategies for health promotion, disease prevention and health protection, as well as the factors that influence the delivery and use of health services.	0	2.9	11.4	60.0	25.7	35
1.2 Demonstrate knowledge about the history, structure and interaction of public health and health care services at local, provincial/territorial, national, and international levels.	0	8.6	31.4	51.4	8.6	35
1.3 Apply the public health sciences to practice.	5.7	5.7	20.0	57.1	11.4	35
1.4 Use evidence and research to inform policies and programs	0	2.9	14.7	44.1	38.2	34
**2. Assessment and Analysis**						
2.1 Recognize that a health concern or issue exists.	0	2.9	2.9	48.6	45.7	35
2.2 Identify relevant and appropriate sources of information including community assets and resources.	2.9	0	5.7	65.7	25.7	35

2.3 Collect, store, retrieve and use accurate and appropriate information on public health issues.	2.9	2.9	11.4	48.6	34.3	35
2.4 Analyze information to determine appropriate implications, uses, gaps and limitations.	0	2.9	22.9	45.7	28.6	35
2.5 Determine the meaning of information considering the current ethical, political, scientific, socio-cultural and economic contexts.	0	8.6	11.4	62.9	17.1	35
2.6 Recommend specific actions based on the analysis of information.	5.7	2.9	22.9	54.3	14.3	35
**3. Policy and Program Planning, Implementation and Evaluation**						
3.1 Describe selected policy and program options to address a specific public health issue.	2.9	2.9	26.5	52.9	14.7	34
3.2 Describe the implications of each option, especially as they apply to the determinants of health and recommend or decide on a course of action.	2.9	2.9	20.6	55.9	17.7	34
3.3 Develop a plan to implement a course of action taking into account relevant evidence, legislation, emergency planning procedures, regulations and policies.	6.1	6.1	36.4	36.4	15.2	33
3.4 Implement a policy or program and/or take appropriate action to address a specific public health issue.	2.9	2.9	58.8	26.5	8.8	34
3.5 Implement effective practice guidelines.	9.1	6.1	45.5	27.3	12.1	33
3.6 Evaluate an action, policy or program.	17.7	2.9	35.3	29.4	14.7	34
3.7 Set and follow priorities, in order to maximize outcomes based on available resources.	5.9	8.8	23.5	52.9	8.8	34

3.8 Fulfill functional roles in response to a public health emergency.	27.3	15.2	21.2	27.3	9.1	33
**4. Partnerships, Collaboration and Advocacy**						
4.1 Identify and collaborate with partners in addressing public health issues.	3.0	0	18.2	33.3	45.5	33
4.2 Use skills such as team building, negotiation, conflict management and group facilitation to build partnerships.	3.1	6.3	18.8	46.9	25.0	32
4.3 Mediate between differing interests in the pursuit of health and well-being, and facilitate the allocation of resources.	6.1	9.1	33.3	36.4	15.2	33
4.4 Advocate for healthy public policies and services that promote and protect the health and well-being of individuals and communities.	3.1	9.4	9.4	53.1	25.0	32
**5. Diversity and Inclusiveness**						
5.1 Recognize how the determinants of health influence the health and well-being of specific populations.	0	2.9	2.9	29.4	64.7	34
5.2 Address population diversity when planning, implementing, adapting and evaluating public health programs and policies.	0	2.9	20.6	32.4	44.1	34
5.3 Apply culturally relevant and appropriate approaches with people from diverse cultural, socioeconomic and educational backgrounds, and persons of all ages, genders, health status, sexual orientations and abilities.	2.9	2.9	20.6	47.1	26.5	34
**6. Communication**						

6.1 Communicate effectively with individuals, families, groups, communities, and colleagues.	0	2.9	0	67.7	29.4	34
6.2 Interpret information for professional, non-professional and community audiences.	0	0	2.9	50.0	47.1	34
6.3 Mobilize individuals and communities by using appropriate media, community resources and social marketing techniques.	0	9.1	15.2	48.5	27.3	33
6.4 Use current technology to communicate effectively.	0	5.9	20.6	35.3	38.2	34
**7. Leadership**						
7.1 Describe the mission and priorities of the public health organization where you work, and apply them in practice.	0	0	13.3	53.3	33.3	30
7.2 Contribute to developing key values and a shared vision in planning and implementing public health programs and policies in the community.	3.0	3.0	18.2	57.6	18.2	33
7.3 Utilize public health ethics to manage yourself, others, information and resources.	2.9	8.8	14.7	52.9	20.6	34
7.4 Contribute to team and organizational learning in order to advance public health goals.	0	5.9	5.9	61.8	26.5	34
7.5 Contribute to maintaining organizational performance standards.	0	3.1	28.1	46.9	21.9	32
7.6 Build community capacity by sharing knowledge, tools, expertise and experience.	2.9	8.8	11.8	44.1	32.4	34
